# Identification of Lower Respiratory Tract Pathogens in Cancer Patients: Insights into Fatal Outcomes

**DOI:** 10.3390/microorganisms12081686

**Published:** 2024-08-16

**Authors:** Dalia F. Mourad, Samah Radwan, Rana Hamdy, Dina M. Elkhashab, Mahmoud M. Kamel, Ahmed S. Abdel-Moneim, Dalia Y. Kadry

**Affiliations:** 1 Clinical Pathology Department, National Cancer Institute, Cairo University, Cairo 11796, Egypt; 2 Pediatric Oncology Department, National Cancer Institute, Cairo University, Cairo 12613, Egypt; 3 Department of Microbiology, College of Medicine, Taif University, Al-Taif 21944, Saudi Arabia

**Keywords:** LRTI, cancer, respiratory viruses, bacteria, antibiotic resistance, FTD-33

## Abstract

This study aimed to investigate LRTIs in cancer patients, focusing on pathogen distribution, and outcomes based on tumor types and antimicrobial treatments. The study included 110 cancer patients exhibiting symptoms of lower respiratory tract infections (LRTIs), consisting of 67 males and 43 females across a wide age range from under 1 year to over 60 years old. Exclusion of SARS-CoV-2 infection was conducted before admission. In addition to classical microbiological methods, fast-track detection using Multiplex Real-Time PCR was employed, utilizing the FTD-33 test kit. The findings revealed a diverse landscape of infections, notably *Klebsiella pneumoniae*, *Haemophilus influenzae* and *Staphylococcus aureus*. Parainfluenza 3 and 4 viruses, rhinovirus, influenza A subtype H1N1pdm09, influenza B and C viruses, HCoV-229, HCoV-OC43, and HCoV-HKU1 were infrequently detected. Furthermore, the existence of mixed infection highlighted the complexity of disease conditions in cancer patients. An analysis of antimicrobial treatment highlighted significant variations in fatal outcomes for carbapenem and colistimethate sodium. It was concluded that mixed infections were commonly identified as potential causes of LRTIs among cancer patients, while viral infections were less frequently detected. It underscores the complexity of antimicrobial treatment outcomes.

## 1. Introduction

Lower respiratory tract infections (LRTIs) stand out as a significant concern that threatens the life of cancer patients. The causative agents of RTIs encompass a diverse range, including bacteria, viruses, and fungi. The compromised immune systems of cancer patients, a consequence of both the disease itself and its accompanying treatment, render them particularly susceptible to respiratory infections. Cancer patients face an elevated risk of contracting pneumonia, primarily attributed to compromised immune function resulting from both the tumor and cancer treatment [1]. In the complex landscape of pathogens contributing to LRTIs, bacteria and viruses take center stage, presenting formidable obstacles to the well-being of individuals grappling with cancer. Factors such as age, lymphopenia, high-dose total body irradiation, and the presence of co-pathogens significantly contribute to LRTI progression [2].

Respiratory viruses are the primary incriminator behind LRTIs [3]. In healthy children, these infections usually result in self-limiting upper respiratory tract infections (URTI) with a mild course. However, individuals with compromised immune systems are more prone to severe infections [4,5,6]. Respiratory viral infections include RNA viruses such as paramyxoviruses (Respiratory syncytial virus (RSV), parainfluenza, Human metapneumovirus (HMPV)), orthomyxoviruses (influenza), picornaviruses (rhinovirus), along with DNA viruses like adenovirus, bocavirus, and polyomaviruses [5,7,8]. RNA viruses are major contributors to respiratory infections in immunocompromised patients [7,8,9]. The severity of the disease depends on viral factors and patient characteristics, with immunosuppression possessing the highest morbidity and mortality [8,10,11]. Immunocompromised patients are significantly more likely to experience adverse outcomes, including pneumonia, respiratory failure, and increased mortality rates, compared to their non-immunocompromised counterparts [6,12]. In adults with cancer, the progression to lower respiratory tract infection (LRTI) ranges from 30 to 50%, with pneumonia-associated mortality as high as 75% [4,9,13,14,15,16]. In children undergoing HCT, mortality typically ranges from 10 to 14%, although it can escalate to 30%, which is significantly higher than in the general population [15,17,18]. Moreover, long-term complications, such as airflow obstruction and bronchiolitis obliterans, have been observed in hematopoietic cell transplantation (HCT) and lung transplant recipients [4,16].

Bacterial pneumonia stands out as the prevailing infection in oncology patients, making them susceptible to acute respiratory failure. Classic community-acquired pathogens, including *Streptococcus pneumonia*, *Staphylococcus aureus*, *Pseudomonas aeruginosa*, *Enterobacter cloacae*, *and Klebsiella pneumonia* are among the common pathogens. *Legionella pneumophila*, *Mycoplasma pneumonia, Coxiella burnetii* and *Chlamydia pneumoniae* are among the intracellular microorganisms detected in pneumonia of cancer patients [19].

Cancer patients face an elevated risk of contracting pneumonia, primarily attributed to compromised immune function resulting from both the tumor and cancer treatment [1]. The treatment approach for community-acquired pneumonia in non-neutropenic cancer patients aligns with general population guidelines [20]. Beta-lactam antibiotics serve as the cornerstone of therapy, with the selection contingent on local epidemiological factors, such as pneumococcal resistance levels. Patients with community-acquired pneumonia require antibiotics covering atypical pathogens like *Mycoplasma pneumoniae, Chlamydophila pneumoniae,* and *Legionella pneumophila* [21]. For those requiring hospitalization, options include respiratory fluoroquinolones or a combination of macrolides with a third-generation cephalosporin (ceftriaxone or cefotaxime) or ertapenem [20]. Ertapenem, in addition to its Gram-positive and Gram-negative activity (excluding *Pseudomonas aeruginosa* and *Acinetobacter* spp.), boasts anaerobic activity, beneficial for suspected aspiration and post-obstructive pneumonia [20,22]. Severe community-acquired pneumonia and pneumonia in neutropenic patients necessitate a combination of antipseudomonal beta-lactam (piperacillin/tazobactam) and respiratory fluoroquinolone or azithromycin [20,21]. For patients with a medical facility background or extensive oncological treatment, and those with frequent hospitalizations, the etiological microorganisms of pneumonia differ. Therefore, the empirical antibiotic choice should encompass *Staphylococcus aureus*, *Pseudomonas aeruginosa* or other Gram-negative rods: piperacillin/tazobactam, cefepime, or carbapenems, combined with antibiotics covering Gram-positive cocci (linezolid, vancomycin, or teicoplanin) [23]. The initial antibiotic regimen selection should be informed by knowledge of local antibiotic susceptibility patterns [20,23]. Clinical samples, including high-quality sputum, lower respiratory tract specimens for culture, viral (influenza, COVID-19), mycoplasma, chlamydial PCR detection, and urine samples for pneumococcal and legionella antigen detection, should precede antibiotic therapy initiation in severe pneumonia cases in immunocompromised patients [24,25]. Microbiological results aid in deescalating and precisely targeting antibiotic treatment [23]. In patients receiving a prednisone equivalent of ≥20 mg for ≥4 months or undergoing radiation therapy with concomitant temozolomide without reliable antipneumocystis prophylaxis, consideration should be given to adding high-dose trimethoprim/sulfamethoxazole [20,21].

The identification of pathogens in laboratory diagnosis for RTIs involves direct methods like culture, rapid antigen testing, or nucleic acid amplification testing (NAAT). This is coupled with clinical and radiological examinations and inflammatory marker laboratory tests. Identifying just one pathogen in a simplex RT–PCR, especially when multiple respiratory viruses require identification, makes the process lengthy, resource-intensive, and costly. Recently, innovative technologies, such as the Respiratory Panel (FA-RP) and batch analysis options like the FTD^®^ Respiratory Pathogens 21 and 33 Panel, have been introduced for detecting multiple targets [26,27].

This study seeks to assess the prevalence of the respiratory pathogens responsible for inducing LRTIs in cancer patients and to screen the most commonly used antibiotics.

## 2. Materials and Methods

### 2.1. Participants

The research obtained approval from the Institutional Review Board of Cairo University National Cancer Institute (Approval No. 2207-310-031, dated 22 July 2022). Before participating, patients or their legal guardians provided informed written consent. A total of 110 participants suspected of lower respiratory tract infection (LRTI) based on clinical or radiological findings were enrolled. Recruitment occurred between 1 April 2023 and the end of August 2023, encompassing inpatient wards across departments such as pediatrics, medical oncology, and surgery at the National Cancer Institute, Cairo University. All participants, Egyptians aged from under 1 year to over 60 years, underwent comprehensive clinical assessments including medical history, physical examinations, primary cancer diagnosis, and antibiotic treatment. LRTI suspicion relied on symptoms like fever, cough, dyspnea, and abnormal chest sounds observed during hospital stays. Diagnostic criteria included chest X-ray and CT scan evidence of consolidations, infiltrates, and other abnormalities consistent with lower respiratory tract infections. In this study, Severe Acute Respiratory Infection (SARI) referred to severe respiratory illnesses necessitating hospitalization, while LRTI specifically targeted infections affecting the lungs’ lower airways, such as pneumonia or bronchitis. Fatal outcomes referred to patients who died during hospitalization due to LRTI complications, while non-fatal outcomes indicated recovery or discharge following treatment. Participants were monitored until hospital discharge or resolution of acute illness, with treatment outcomes documented. Management protocols followed Infectious Diseases Society of America (IDSA) guidelines, including antibiotic initiation for high-risk febrile neutropenia and antifungal therapy if the fever persisted beyond day 5. Acyclovir was administered to patients reporting dysphagia or herpetic oral ulcers.

### 2.2. Sample Collection and Processing

Nasopharyngeal swabs were obtained primarily from children, whereas adults were sampled with both nasopharyngeal swabs and sputum within 24 h of the onset of symptoms from the affected patients with community acquired Severe Acute Respiratory Infection (SARI), an acute respiratory illness accompanied by fever (≥38 °C) and cough, typically necessitating hospitalization, within the initial 24 h of symptoms. Samples to be tested by the Fast Track Detection technique were combined into a single tube and were promptly stored at 4 °C for temporary storage. NPS samples were stored at −20 °C until the testing process could be conducted. The second sample (swab or sputum) was immediately processed for the detection of causative organisms using conventional microbiological methods for bacteria, as well as fungal culture on *Sabouraud* dextrose agar. This was followed by the Fast Track Detection technique that employed Multiplex Real-Time PCR for the simultaneous detection of viral, bacterial, and fungal infections. Blood samples were used for screening routine hematological and biochemical biomarkers.

### 2.3. Nucleic Acid Extraction

For the preliminary extraction procedure, the VERSANT^®^ Sample Preparation 1.0 Reagents kit was employed on an automated APEX KING FISHER platform by Thermofisher Scientific (Waltham, MA, USA). The process involved handling 96 samples per run, with an extraction starting volume of 400 μL and an elution volume of 110 μL.

### 2.4. SARS-CoV-2 Screening

SARS-CoV-2 screening is conducted for every patient admitted to NCI clinics through a rapid antigen test. Those testing positive subsequently undergo a confirmatory RT-qPCR test. Only patients with negative test results are permitted admission for chemotherapy sessions, clinics, or inpatient care. Individuals testing positive for SARS-CoV-2 are isolated in designated wards until their recovery, in accordance with guidelines outlined by the World Health Organization (WHO).

### 2.5. Real-Time PCR for Detection of Respiratory Pathogens

Genomic RNA obtained from respiratory specimens underwent reverse transcription using specific primers, followed by immediate polymerase chain reaction (PCR) in the same tube. Simultaneously, the DNA of various pathogens was amplified in the same tube through PCR. Equine arteritis virus (EAV) served as an internal control (IC) and was introduced into each sample and the negative control during the extraction process. The detection of respiratory pathogens involved real-time reverse-transcriptase polymerase chain reactions (rRT-PCR) using the FTD-33 Test Kit (Fast Track Diagnostics, Esch-sur-Alzette, Luxembourg). The panel encompasses 12 bacterial targets, 20 viral targets, and 1 fungal target (*P. jirovecii*). The bacterial targets include H. influenzae, Bordatella species (excluding *B. parapertussis*), *M. catarrhalis*, Salmonella species, *L. pneumophilia/longbeachiae, K. pneumoniae, S. aureus, S. pneumoniae, C. pneumoniae*, and *M. pneumoniae*. On the viral front, the targets consist of influenza A, B, C, then subtype H1N1pdm09, rhinovirus, coronaviruses (NL63, 229E, OC43, HKU1), parainfluenzaviruses (1–4), metapneumoviruses (A and B), bocavirus, hRSV serotypes (A and B), human adenovirus, enterovirus, and parechovirus. Eight multiplex rRT-PCR reactions adhered to the manufacturer’s instructions. Each reaction mix comprised 10 µL TNA, 1.5 µL of oligonucleotide mix, 12.5 µL 2X RT- PCR buffer (Fast-track mastermix), and 1 µL 25 X RT-PCR Enzyme Mix (Fast- track mastermix). Each specimen was tested for the presence of human ribonuclease P (RNase P), serving as a control for extraction and PCR inhibition. All experiments were conducted using the QuantStudioᵀᴹ 5 Real-Time PCR, employing the subsequent cycling parameters: initial reverse transcription for RNA viruses at 50 °C for 15 min, hold, initial denaturation for 94 °C for 1 min, hold, followed by 40 cycles at 94 °C for 8 s, and 60 °C for 60 s.

### 2.6. Statistical Analysis

The statistical analysis included various techniques in SPSS. Descriptive statistics were employed to provide a detailed summary of the key characteristics of the dataset. Crosstabs were utilized to investigate associations and patterns within the data. The Chi-square test was applied to assess the association between categorical variables and determine if there were significant relationships among them. Furthermore, the Kruskal–Wallis test was employed to examine potential variations among different groups. A significance level of *p* < 0.05 was considered statistically significant.

## 3. Results

### 3.1. Tumor Distribution and Fatal Consequence of the Patients

The study included 110 participants, with a gender distribution of 43 (39.1%) females and 67 (60.9%) males. The analysis revealed distinct patterns in the occurrence and fatality rates across various age groups and types of tumors. Solid tumors, lymphomas, leukemias, and other hematological malignancies accounted for 42 (38.2%), 15 (13.6%), 50 (45.5%), and 3 (2.7%) of the cases, respectively (Table 1). The distribution of solid tumors among patients encompassed a range of cancer types: adenocarcinoma (two cases), anaplastic oligodendroglioma (one case), primitive neuro-ectodermal tumors (one case), mucinous carcinoma (one case), thymoma (one case), bronchogenic carcinoma (three cases), Pancreatic cancer (one case), rectal cancer (three cases), cholangiocarcinoma (two cases), chondrosarcoma (one case), choroid plexus carcinoma (one case), endometrial carcinoma (two cases), esophageal cancer (one case), urothelial carcinoma (two cases), squamous cell carcinoma (six cases), juvenile xanthogranuloma (one case), medulloblastoma (one case), neuroblastoma (three cases), laryngeal squamous cell carcinoma (two cases), neuroendocrine tumor (one case), Wilms tumor (one case), sarcoma (three cases), renal cell carcinoma (one case), and dysgerminoma (one case).

In the age group of 0–12 years, notable fatal cases were observed, with 11 out of 32 resulting in fatality. This included a nuanced distribution across genders and specific tumor types, with 4/11 (36.3%) of fatal cases attributed to solid tumors, 4/11 (36.3%) to lymphomas, and 3/11 (27.3%) to leukemias. For the 13–17 age group, the study reported nine cases, with five fatalities. The fatalities included a mix of genders and specific tumor types, with 5/5 (100%) attributed to leukemias (Table 1). The young adult group (18–40 years) exhibited 21 cases, of which 7 were fatal. The fatalities were predominantly associated with leukemias, 4/13 (30.8%), emphasizing the severity in this age range. Non-fatal cases included lymphomas and other tumors. The older adult group (41–60 years) comprised 34 patients, with a notable distribution of solid tumors, 15/34 (44.1%), and leukemias, 15/34 (44.1%). In elderly adults (>60 years), 14 patients were included, primarily with solid tumors, 12/14 (85.7%). The fatality rate in patients with solid tumors in this group was 6/12 (50%) and 2/4 (50%) in leukemia patients (Table 1). Of the solid tumors, 17/42 (40.5%) were fatal, while leukemias demonstrated 20/49 (40.8%) fatal cases.

### 3.2. Causative Agents of Lower Respiratory Tract Infections in Cancer Patients Based on Real-Time PCR

*Klebsiella pneumoniae* infections were more prevalent, particularly in certain age groups including nine cases in 41–60 years, eight cases in age 0–12 years, and six cases in >60. *Klebsiella pneumoniae* contributed to a total of 9 non-fatal and 17 fatal cases (Table 2). *Staphylococcus aureus* infections demonstrated a relatively balanced distribution, with a total of 12 non-fatal and 11 fatal cases. *Haemophilus influenzae* was confirmed in five non-fatal and two fatal cases, playing a varied distribution across age groups. *Streptococcus pneumoniae* exhibited a balanced distribution, with one non-fatal and one fatal case. *Legionella pneumonia*, however, presented only fatal cases, indicating a severe outcome in the reported instances. *Moraxella catarrhalis* infections were limited, with two non-fatal cases. *Pneumocystis jiroveci* pneumonia showed an equal distribution with one non-fatal and one fatal case. *Rhinovirus* infections were reported in six male patients only, including five cases within the age range of 0–12 years (two of them were fatal) and the 6th case was fatal among patients within the age group of 13–17 years (Table 2). Influenza B virus and Influenza A virus H1N1pdm09 showed limited occurrences, with one non-fatal case each. Whereas influenza C virus was reported in a single fatal case aged from 0–12 years. Out of the nine infections with human coronaviruses, five were fatal including *one out* of three who were infected with HCoV-*229*, three out of four cases infected with HCoV-OC43, and one out of two cases infected with HCoV-KHU1. Parainfluenza viruses (PIV-3 and PIV-4) were reported in nine cases across different age groups: non-fatal cases were reported in three PIV-3 cases while fatal cases were reported in four cases infected with PIV-3 and two cases infected with PIV-4 (Table 2).

### 3.3. Respiratory Infections Categorized by the Type of Tumors in the Studied Population

*Klebsiella pneumoniae* exhibited nine non-fatal cases, including six in patients with solid tumors, a single lymphoma case, and two leukemia patients. Additionally, 17 fatal cases were reported, with 10 occurring in patients with solid tumors, 4 in patients with leukemia, 1 in a lymphoma patient, and 2 in patients with other tumors. *Staphylococcus aureus* was detected in 23 patients, including 11 with leukemia (5 out of 11 were fatal), 8 with solid tumors (50% were fatal), 2 with lymphoma (resulting in a single fatal case), and 2 with other tumors (each with a single fatal case). *Haemophilus influenzae* was found in seven patients: three with solid tumors, three with leukemia, and a single lymphoma patient. Fatal cases were reported in two out of seven patients (one with a solid tumor and the other with leukemia). *Streptococcus pneumoniae* was identified in two cases, a single non-fatal case in a lymphoma patient and a single fatal leukemia patient. *Legionella pneumonia* was detected in a single fatal case in a leukemia patient, and *Moraxella catarrhalis* in two non-fatal cases (one with a solid tumor and one with lymphoma).

Rhinovirus was detected in three leukemia patients, with one out of the three being fatal. It was also detected in two cases of solid tumor (one of them fatal) and one fatal lymphoma patient. Influenza A virus H1N1pdm09 was found in a non-fatal case in solid tumors, while influenza B virus was identified in a single non-fatal case in leukemia. Influenza C virus was detected in a single patient with a solid tumor. Nine cases of coronavirus infection were detected, including three fatal cases by HCoV-OC43 in a single case of solid tumor, leukemia, and other tumors. A single fatal case out of three infections reported by HCoV-229 occurred in solid tumor, while other non-fatal infections by this virus were detected in patients with solid tumors and lymphoma. Meanwhile, HCoV-KHU1 infections resulted in a non-fatal infection in a patient with solid tumor and a fatal infection in a single patient with leukemia. *PIV-3* was detected in four patients with solid tumors and two patients with leukemia (50% of them were fatal). Additionally, PIV-4 infection resulted in two fatal infections in one patient with lymphoma and the other with leukemia (Table 3).

### 3.4. Mixed Infection Screening in Cancer Patients with LRTI

Upon analyzing cultural and real-time PCR results, a total of 12 cancer patients did not exhibit bacterial or fungal growth or test positive for the screened viruses, including 3 fatalities and 9 classified as non-fatal. The specific microorganism remains unidentified. In 16 cases, mixed viral and bacterial infections were observed, resulting in 8/16 (50%) fatal cases. Mixed viral, bacterial, and fungal infections were noted in two non-fatal cases. Mixed bacterial infections were identified in 12 cases, with 3 non-fatal cases and 9 fatal cases. Additionally, mixed bacterial and fungal infections encompassed 13 cases, leading to 4 fatalities and 9 non-fatal cases. A single infection was reported with H1N1pdm09, providing insights into its varied impacts in different contexts (Table 4).

*Staphylococcus aureus*, MRSA, *Klebsiella pneumoniae*, coagulase-negative *Staphylococci* (CONS), *E. coli, Proteus* spp., *Pseudomonas*, *Candida* spp., and *Pneumocystis jirovecii*—each of these microorganisms exhibited distinct fatality and non-fatality patterns.

### 3.5. Use of Antimicrobials in the Treatment of Cancer Patients with LRTI

Carpapenem is the most frequently administered antimicrobial, totaling 75 instances. Colistimethate soduim follows this with 39 occurrences. Vancomycin, tigecycline, aminoglycoside, and pipracillin/tazobactam were also frequently used, with 34, 28, 39, and 25 occurrences, respectively. Caspofungin, an antifungal, was used in 26 cases, although fungal infections were identified in only 18 cases, including mixed infections. Conversely, less frequent use was observed for flouroquinoloe, third-generation chephalosporin (ceftriaxone, cefipime) sulfamethoxazole/trimethoprim, fluoroquinolone, linezolid, and amoxacillin/clavulonic acid. Eleven patients with solid tumors have been treated with amoxacillin/clavulonic acid, with eight out of eleven non-fatal infections and three out of eleven fatal infections (*p* < 0.001). Vancomycin was significantly utilized in 24 leukemia patients, and half of them died (*p* < 0.001). In contrast, ceftriaxone, a third-generation cephalosporin, was employed in six patients with solid tumors and resulted in 2/6 (33.3% death) (*p* < 0.009). Amikacin demonstrated notable impact, particularly in leukemia cases, being administered in 28 patients from whom 13/28 died (*p* < 0.001).

The Kruskal–Wallis test results examining fatal consequences following antimicrobial treatments showed significant variations for carbapenem (*p* = 0.013) and colistimethate soduim (*p* = 0.000). In contrast, other treatments, including vancomycin, tigecycline, flouroquinolone, third-generation cephalosporin (ceftriaxone, cefipime) piperacillin//tazobactam, sulfamethoxazole and trimethoprim, amikacin, levofloxacin, linezolid, amoxacillin/clavulonic acid and caspofungin (antifungal), exhibited no statistically significant differences (Table 5).

## 4. Discussion

Cancer, often associated with immunosuppression due to both the disease and its treatments, renders individuals more susceptible to infections. Respiratory infections, ranging from viral to bacterial etiologies, can lead to severe complications in both young and adult populations [28,29]. Bacterial pneumonia, a common and potentially life-threatening complication, significantly contributes to the global burden of respiratory infections in cancer patients. Pathogens like *Streptococcus pneumoniae*, *Haemophilus influenzae*, and *Staphylococcus aureus* often play a role in pneumonia cases, further complicating the clinical course in cancer patients [30,31]. In a study involving 18,807 community-acquired pneumonia (CAP) patients, 20.05% had severe CAP (SCAP). Children (≤5 years) and older adults (>60 years) with SCAP exhibited higher rates of viral and bacterial infections, including viral–bacterial co-infections. In contrast, adults aged 18–60 years with SCAP showed a higher rate of bacterial–bacterial co-infection. Predominant pathogens varied by age, with RSV and *S. pneumoniae* common in children, while influenza virus and *P. aeruginosa* were prevalent in older adults. Human adenovirus, human rhinovirus, and *Klebsiella pneumoniae* in children, and *P. aeruginosa, K. pneumoniae*, or *S. pneumoniae* in adults, emphasizing age-specific microbial patterns in severe pneumonia cases [29]. Bacterial pneumonia is a prevalent infection leading to acute respiratory failure in oncology patients. Among 424 cancer patients experiencing respiratory failure with known causes, 47% were identified with bacterial pneumonia. Streptococcus pneumoniae was the most common community-acquired pathogen (25%), followed by other streptococci. Healthcare-associated organisms, including Staphylococcus aureus, *Pseudomonas aeruginosa, Enterobacter cloacae, and Klebsiella pneumoniae*, were frequently identified, with varying degrees of virulence and resistance. Additionally, 10% of culture-positive pneumonia cases were linked to intracellular organisms such as *Legionella pneumophila, Mycoplasma pneumoniae, Coxiella burnetii,* and *Chlamydia pneumoniae* [19]. In contrast, the current study showed that *Klebsiella pneumoniae* infections, a more prevalent bacterium among cancer patients with LRTIs, contributed to 9 non-fatal and 17 fatal cases. This was followed by *Staphylococcus aureus*, which had a balanced distribution with 12 non-fatal and 11 fatal cases. Conversely, *Haemophilus influenzae* was confirmed as the major cause in five non-fatal and two fatal cases.

In cancer patients, pneumonia is often complicated by mixed infections, involving various combinations of bacteria, viruses, and fungi. These interactions can contribute to the severity of the disease and pose challenges for effective treatment [32,33,34]. In the current study, an equal number of fatalities and non-fatal outcomes was observed in eight cases with mixed viral and bacterial infections which underscores the severity of such combinations. The complexity further intensifies in two non-fatal cases with mixed viral, bacterial, and fungal infections, indicating a higher level of clinical intricacy.

In immunocompromised individuals, such as cancer patients, isolation of CoNS or Candida may indicate underlying lung disease or a compromised immune response. CoNS and Candida are also associated with hospital-acquired infections. Candida species are among the leading pathogens responsible for healthcare-associated bloodstream infections, alongside *Staphylococcus aureus*, coagulase-negative staphylococci, and *Enterococcus* species [35]. Isolation of these organisms from lower respiratory samples may co-exist with other pathogens in polymicrobial infections, complicating diagnosis and outcome of infection [35,36].

The consequences of bacterial interactions are evident in the mixed bacterial infections, with nine fatalities out of twelve instances. Moreover, two non-fatal cases with mixed viral, bacterial, and fungal infections emphasized the complexity, highlighting intricate clinical scenarios. We hypothesize that mixed viral and bacterial infections could provoke heightened immune responses and synergistic pathogenic interactions, potentially leading to more severe clinical outcomes compared to infections caused by a single pathogen. This underscores the unique challenges posed by mixed infections in immunocompromised cancer patients, where multiple pathogens may collaboratively compromise respiratory function. The consequences of bacterial interactions are evident in mixed bacterial infections, where nine out of twelve cases resulted in fatalities. This underscores the diverse and sometimes contrasting outcomes associated with bacterial coexistence. Moreover, the thirteen cases involving mixed bacterial and fungal infections contributed to a notable impact on patient outcomes, with four fatalities and nine non-fatal cases.

In the current study, vancomycin was significantly used in leukemia patients, particularly those with fatal cases. Ceftriaxone, a third-generation cephalosporin was administered exclusively in patients with solid tumors, and amikacin usage was predominant in leukemia cases. Amoxicillin/clavulanic acid was predominantly used in patients with solid tumors who showed a high rate of non-fatal infection. Carbapenem was notably more frequently administered in fatal cases, particularly in instances involving *Klebsiella pneumoniae, Staphylococcus aureus*, and *Legionella pneumophila* infections. Interestingly, it was also used in the case where the patient was infected with *Pneumocytis jiroveci*. Colistimethate sodium was effective in treating non-fatal cases; however, it was notably more commonly used in fatal cases associated with various pathogens.

A clear finding demonstrated that antimicrobial treatments with carbapenem colistimethate sodium showed significant associations. These findings emphasize the importance of antibiotic selection in influencing treatment outcomes. Carbapenem and Colistimethate sodium play crucial roles in treating severe bacterial infections, especially pneumonia. Carbapenem, a broad-spectrum carbapenem, is effective against various bacteria but faces concerns about resistance and requires careful use based on susceptibility and clinical context. Colistimethate sodium, a polymyxin for multidrug-resistant Gram-negative bacteria, has seen renewed use but is linked to nephrotoxicity and neurotoxicity. The choice of these antibiotics should consider patient demographics, pathogen types, and underlying health conditions, emphasizing the need for judicious use in the face of antibiotic resistance [37,38].

The study’s limitations include heterogeneous cancer types among participants and the absence of an asymptomatic cancer control group. Virus identification primarily relied on upper respiratory specimens like nasopharyngeal aspirates or swabs, potentially detecting coincidental upper respiratory infections. Not all pneumonia cases underwent comprehensive pathogen testing, limiting pathogen identification. Follow-up sampling to differentiate prolonged versus persistent shedding of detected respiratory viruses was lacking. Lastly, the study’s single-center design restricts generalization of the findings.

## 5. Conclusions

This study offers novel insights into the complex interplay between respiratory infections, tumor types, and antimicrobial treatments in a cohort of 110 cancer patients. The research reveals distinct patterns in fatality rates across various age groups and tumor categories, pinpointing specific vulnerabilities, especially among leukemia patients. The identification of mixed infections and their association with severe outcomes provides a nuanced understanding of the complex nature of respiratory infections in cancer patients.

## Figures and Tables

**Table 1:** Distribution of tumors and fatality based on age and sex.

Age (Yr)	Fatality	Sex			Type of Tumor				Total
		Female	Male		Solid Tumor	Lymphoma	Leukemia	Other Tumors	
0–12	Non-Fatal	4 (19.0)	17 (81.0)		6 (28.6)	4 (19.0)	11 (52.4)	0 (0.0)	21 (65.6)
	Fatal	3 (27.3)	8 (72.7)		4 (36.3)	4 (36.3)	3 (27.3)	0 (0.0)	11 (34.4)
	Total	7 (21.9)	25 (78.1)		10 (31.3)	8 (25.0)	14 (43.8)	0 (0.0)	32 (29.1)
13–17	Non-Fatal	2 (50.0)	2 (50.0)		2 (50.0)	1 (25.0)	1 (25.0)	0 (0.0)	4 (44.4)
	Fatal	3 (60.0)	2 (40.0)		0 (0.0)	0 (0.0)	5 (100.0)	0 (0.0)	5 (55.6)
	Total	5 (55.6)	4 (44.4)		2 (22.2)	1 (11.1)	6 (66.7)	0 (0.0)	9 (8.2)
18–40	Non-Fatal	6 (42.9)	8 (57.1)		3 (21.4)	2 (14.3)	9 (64.3)	0 (0.0)	14 (66.7)
	Fatal	4 (57.1)	3 (42.9)		0 (0.0)	1 (14.3)	4 (57.1)	2 (28.6)	7 (33.3)
	Total	10 (57.6)	11 (52.4)		3 (14.3)	3 (14.3)	13 (61.9)	2 (9.5)	21 (20.0)
41–60	Non-Fatal	8 (42.1)	11 (57.9)		8 (42.1)	3 (15.8)	8 (42.1)	0 (0.0)	19 (55.9)
	Fatal	6 (40.0)	9 (60.0)		7 (46.7)	0 (0.0)	7 (46.7)	1 (6.6)	15 (44.1)
	Total	14 (41.2)	20 (58.8)		15 (44.1)	3 (8.8)	15 (44.1)	1 (2.9)	34 (30.9)
>60	Non-Fatal	2 (33.3)	4 (66.7)		6 (100.0)	0 (0.0)	0 (0.0)	0 (0.0)	6 (42.9)
	Fatal	5 (62.5)	3 (37.5)		6 (75.0)	0 (0.0)	2 (25.0)	0 (0.0)	8 (57.1)
	Total	7 (50.0)	7 (50.0)		12 (85.7)	0 (0.0)	2 (14.3)	0 (0.0)	14 (12.7)
Cumulative		43(39.1)	67 (60.1)		42 (38.2)	15 (13.6)	50 (45.5)	3 (2.7)	110

Note: Percentages are in parentheses.

**Table 2:** Prevalence of various viral and bacterial pathogens in cancer patients with lower respiratory tract infections.

Pathogen ^a^	Fatality	Sex		Age					Total
		Female	Male	0–12 yr	13–17 yr	18–40 yr	41–60 yr	>60	
*Klebsiella pneumoniae*	Non-fatal	1	8	4	0	0	3	2	9
	Fatal	10	7	4	1	2	6	4	17
*Staphylococcus aureus*	Non-fatal	4	8	2	2	5	3	0	12
	Fatal	6	5	3	2	3	0	3	11
*Haemophilus influenzae Disease (Including Hib)*	Non-fatal	2	3	1	0	2	2	0	5
	Fatal	0	2	0	1	0	0	1	2
*Streptococcus pneumoniae*	Non-fatal	0	1	0	0	1	0	0	1
	Fatal	1	0	0	0	0	1	0	1
*Legionella pneumonia*	Non-fatal	0	0	0	0	0	0	0	0
	Fatal	0	1	0	0	0	1	0	1
*Moraxella catarrhalis*	Non-fatal	0	2	1	0	0	0	1	2
	Fatal	0	0	0	0	0	0	0	0
*Pneumocystis jiroveci pneumonia*	Non-fatal	0	1	0	0	1	0	0	1
	Fatal	1	0	0	0	0	1	0	1
Rhinovirus	Non-fatal	0	3	3	0	0	0	0	3
	Fatal	0	3	2	1	0	0	0	3
Influenza A virus H1N1pdm09	Non-fatal	1	0	0	0	1	0	0	1
	Fatal	0	0	0	0	0	0	0	0
Influenza B virus	Non-fatal	0	1	0	0	0	0	1	1
	Fatal	0	0	0	0	0	0	0	0
Influenza C virus	Non-fatal	0	0	0	0	0	0	0	0
	Fatal	0	1	1	0	0	0	0	1
HCoV-229	Non-fatal	1	1	1	0	0	1	0	2
	Fatal	0	1	0	0	0	0	1	1
HCoV-OC43	Non-fatal	0	1	0	0	0	0	0	1
	Fatal	2	1	0	1	0	1	1	3
HCoV-KHU1	Non-fatal	1	0	0	0	0	0	1	1
	Fatal	0	1	0	0	0	1	0	1
PIV-3	Non-fatal	1	2	2	0	0	0	1	3
	Fatal	1	3	3	0	0	1	0	4
PIV-4	Non-fatal	0	0	0	0	0	0	0	0
	Fatal	1	1	1	0	1	0	0	2

^a^ Respiratory pathogens were identified utilizing the FTD-33 diagnostic kit. Only pathogens yielding positive results were included in the table above, while those in the FTD-33 kit that did not exhibit positive results were not listed.

**Table 3:** Prevalence of various viral and bacterial pathogens in cancer patients with lower respiratory tract infections classified based on the type of cancer.

Pathogen ^a^	Fatality	Cancer Patients				Total (N = 110)
		Solid Tumor (N = 42)	Lymphoma (N = 15)	Leukemia (N = 50)	Other Tumors (N = 3)	
*Klebsiella pneumoniae*	Non-fatal	6	1	2	0	9
	Fatal	10	1	4	2	17
*Staphylococcus aureus*	Non-fatal	4	1	6	1	12
	Fatal	4	1	5	1	11
*Haemophilus influenzae*	Non-fatal	2	1	2	0	5
	Fatal	1	0	1	0	2
*Streptococcus pneumoniae*	Non-fatal	0	1	0	0	1
	Fatal	0	0	1	0	1
*Legionella pneumonia*	Non-fatal	0	0	0	0	0
	Fatal	0	0	1	0	1
*Moraxella catarrhalis*	Non-fatal	1	1	0	0	2
	Fatal	0	0	0	0	0
*Pneumocystis jiroveci pneumonia*	Non-fatal	0	1	0	0	1
	Fatal	0	0	1	0	1
Rhinovirus	Non-fatal	1	0	2	0	3
	Fatal	1	1	1	0	3
Influenza A virus H1N1pdm09	Non-fatal	1	0	0	0	1
	Fatal	0	0	0	0	0
Influenza B virus	Non-fatal	0	0	1	0	1
	Fatal	0	0	0	0	0
Influenza C virus	Non-fatal	0	0	0	0	0
	Fatal	1	0	0	0	1
HCoV-229	Non-fatal	1	1	0	0	2
	Fatal	1	0	0	0	1
HCoV-OC43	Non-fatal	1	0	0	0	1
	Fatal	1	0	1	1	3
HCoV-KHU1	Non-fatal	1	0	0	0	1
	Fatal	0	0	1	0	1
PIV-3	Non-fatal	2	0	1	0	3
	Fatal	2	1	1	0	4
PIV-4	Non-fatal	0	0	0	0	0
	Fatal	0	1	1	0	2

^a^ Respiratory pathogens were identified utilizing the FTD-33 diagnostic kit. Only pathogens yielding positive results were included in the table above, while those in the FTD-33 kit that did not exhibit positive results were not listed.

**Table 4:** Mixed infection screening in cancer patients with lower respiratory tract infections.

Pathogen	Fatality	Cancer Patients				Total
		Solid Tumor (N = 42)	Lymphoma (N = 15)	Leukemia (N = 50)	Other Tumors (N = 3)	
Not Known	Non-fatal	3	0	6	0	9
	Fatal	1	0	1	1	3
Mixed viral and bacterial infections	Non-fatal	5	1	2	0	8
	Fatal	3	1	3	1	8
Mixed viral, bacterial and fungal infections	Non-fatal	2	0	0	0	2
	Fatal	0	0	0	0	0
Mixed bacterial infections	Non-fatal	1	1	1	0	3
	Fatal	3	1	5	0	9
Mixed bacterial and fungal infections	Non-fatal	2	3	4	0	9
	Fatal	3	0	1	0	4
H1N1pdm09	Non-fatal	1	0	0	0	1
	Fatal	0	0	0	0	0
*Staph. aureus*	Non-fatal	1	0	2	1	4
	Fatal	0	0	0	1	1
*MRSA* ^a^	Non-fatal	1	0	4	0	5
	Fatal	1	1	3	0	5
*Klebsiella pneumoniae*	Non-fatal	2	0	2	0	4
	Fatal	4	1	3	1	9
*CONS*	Non-fatal	5	2	8	0	15
	Fatal	2	0	2	0	4
*E.coli*	Non-fatal	0	0	0	0	0
	Fatal	0	0	2	0	2
*Proteus vulgaris*	Non-fatal	1	0	0	0	1
	Fatal	0	0	0	0	0
*Pseudomonas aeruginosa*	Non-fatal	0	0	0	0	0
	Fatal	0	1	0	0	1
*Candida albicans*	Non-fatal	0	2	0	0	2
	Fatal	0	0	0	0	0
*Pneumocytis jirovecii*	Non-fatal	1	2	0	0	3
	Fatal	0	0	0	0	0

^a^ Methicillin-resistant *Staphylococcus aureus* (*MRSA*). Infections were screened using real-time PCR and classical bacteriological screening of bacteria and fungi.

**Table 5:** Antimicrobial treatments and fatal outcomes in the management of lower respiratory tract infections among patients with various types of cancer.

Antimicrobial	Fatality	Cancer Patients				Total	Cumulative
		Solid Tumor	Lymphoma	Leukemia	Other Tumors		
Carpapenem	Non-fatal	11	5	21	0	37	75
	Fatal	13	4	19	2	38	
Colistimethate Soduim	Non-fatal	4	3	7	0	14	39
	Fatal	7	3	14	1	25	
Aminoglycoside	Non-fatal	4	2	15	0	21	39
	Fatal	5	0	13	0	18	
Vancomycin	Non-fatal	3	2	12	0	17	34
	Fatal	1	3	12	1	17	
Tigacil	Non-fatal	5	2	5	0	12	28
	Fatal	5	2	8	1	16	
Piracillin/Tazobactam	Non-fatal	4	3	9	0	16	25
	Fatal	3	1	4	1	9	
Fluoroquinolone	Non-fatal	7	2	3	0	12	23
	Fatal	4	2	4	1	11	
Sutrium sulfamethoxazole and trimethoprim	Non-fatal	3	1	4	0	8	14
	Fatal	1	0	4	1	6	
Linezolid	Non-fatal	3	1	2	0	6	14
	Fatal	4	2	2	0	8	
Amoxacillin/clavulonic acid	Non-fatal	8	1	1	0	10	14
	Fatal	3	0	0	1	4	
Zithromax	Non-fatal	2	0	1	0	3	6
	Fatal	2	0	0	1	3	
Ceftriaxone third-generation chephalosporin	Non-fatal	4	0	0	0	4	6
	Fatal	2	0	0	0	2	
Cefepime third-generation chephalosporin	Non-fatal	1	0	1	0	2	3
	Fatal	1	0	0	0	1	
Caspofungin (antifungal)	Non-fatal	4	4	10	0	18	26
	Fatal	2	2	4	0	8	

Kruskal–Wallis test showed that both carbapenem (*p* = 0.013) and colistin (*p* = 0.000) significantly affect the fatal consequences. It also showed that vancomycin (*p* < 0.001), third-generation cephalosporin, *third-generation chephalosporin* ceftriaxone (*p* < 0.009), amikacin (*p* < 0.001), *and* amoxicillin/clavulanic acid (*p* < 0.001) were found to be significantly used in certain types of tumors.

## Data Availability

Dataset available upon request from the authors. The raw data supporting the conclusions of this article will be made available by the authors upon request.
